# The complete chloroplast genome of *Paphiopedilum tranlimianum* (Orchidaceae)

**DOI:** 10.1080/23802359.2018.1483779

**Published:** 2018-07-27

**Authors:** Changhai Sui, Shuying Liu, Huimin Liu, Hongzhang Liu

**Affiliations:** a College of Life sciences, Jilin Agricultural University, Changchun City, P. R. China;; b Jilin Engineering Vocational college, Siping City, Jilin, P.R. China

**Keywords:** Paphiopedilum tranlimianum, Illumina sequencing, chloroplast genome, MITObim

## Abstract

Chloroplast (cp) genome sequences have become a useful tool for phylogeneitc and evolutionary study in recent reports. Here the complete chloroplast genome of the *Paphiopedilum tranlimianum* has been reconstructed from the whole-genome Illumina sequencing data. The circular genome is 162,127 bp in size, and comprises of a pair of inverted repeat (IR) regions of 31,457 bp each, a large single-copy (LSC) region of 91,711 bp, and a small single-copy (SSC) region of 7,502 bp. The total GC content is 35.4%, while the corresponding values of the LSC, SSC, and IR regions are 32.7%, 26.9%, and 40.9%, respectively. The chloroplast genome contains 133 genes, including 87 protein-coding genes, eight ribosomal RNA genes, and 38 transfer RNA genes. Three genes (*ndhA*, *ndhE,* and *ndhI*) are pseudogenized or lost in its cpDNA. The Maximum-Likelihood phylogenetic analysis showed a close relationship with *P. niveum* in Orchidaceae. Our findings provide useful information for phylogenetic and evolutionary research of *Paphiopedilum* species.

Chloroplasts are the unique organelles which have their self-replication genomes and are capable to carry out photosynthesis and synthesize starch, fatty acids, and other proteins (Ohyama et al. 1986; Bausher et al. 2006). Because of which the chloroplast genome sequences are a reliable tools for phylogeneitc and evolutionary research, lots of chloroplasts genomes of valuable plants have been reported recently (Jing et al. 2016; Chen et al. 2018).

*Paphiopedilum tranlimianum* (Orchidaceae), native to Vietnam and cultivated artificially in the regions of southern China, like Yunnan, Guizhou, *etc.,* is one of the earliest cultivated orchids in the world for its unique flower shapes and color. To facilitate its genetic research and contribute to its utilization, in this study, we assembled its chloroplast genome using high-throughput Illumina sequencing technology and analyzed its phylogenetic evolution, which will be helpful for further studies on its chloroplast genetic engineering.

DNA samples were extracted from the fresh leaves collected from a single *P. tranlimianum* plant, in Kunming, Yunnan Province and were stored in our lab. The whole genome shotgun sequencing of *P. tranlimianum* was performed by Beijing Gene Insititution (BGI, Shenzhen, China) using the Illumina HiSeq 2000 platform (Illumina, Hayward, CA). Total 24.5 M 125 bp raw reads were retrieved and trimmed by CLC Genomics Workbench v8.0 (CLC Bio, Aarhus, Denmark). A subset of 18.9 M trimmed reads were used for reconstructing the chloroplast genome by MITObim v1.8 (Hahn et al. 2013), with that of its congener *Paphiopedilum armeniacum* (GenBank: NC_026779.1) as the initial reference genome. A total of 16,324,608 individual chloroplast reads yielded an average coverage of 496.7-fold. The chloroplast genome was annotated in GENEIOUS R11 (Biomatters Ltd., Auckland, New Zealand) and was drawn to the circular chloroplast genome sequence map of OGDRAW.

The chloroplast genome of *P. tranlimianum* is a double-stranded circular DNA molecule which is 162,127 bp in size (MH150886). It comprises of a pair of inverted repeat (IR) regions of 31,457 bp each, separated by a large single-copy (LSC) region of 91,711 bp, and a small single-copy (SSC) region of 7,502 bp. The total GC content is 35.4%, while the corresponding values of the LSC, SSC, and IR region are 32.7%, 26.9%, and 40.9%, respectively.

This chloroplast genome harbors 133 functional genes, including 87 protein-coding genes (PCGs), 38 tRNA genes, and eight rRNA genes. 37 PCGs, 16 tRNA genes, and 4 rRNA genes are located in the forward strand while others are located in the reverse strand. Among them, 38 genes are involved in photosynthesis, six genes are in substance metabolism and 30 genes are related with self-replication. Three genes that are *ndhA*, *ndhE,* and *ndhI* are pseudogenized or lost in its cpDNA when compared with that of a closed species, *P. dianthum* and *P. armeniacum* (Hou et al. 2017; Liu et al. 2006).

The Maximum-Likelihood phylogenetic tree was generated using 39 shared PCGs among 37 chloroplast sequences in Orchidaceae by MEGA 6.0 (Tamura et al. 2013), which showed the position of *P. tranlimianum* was situated as the sister of *P. niveum* in Orchidaceae (Figure 1). Our findings will provide a foundation for further investigation of chloroplast genome evolution in *Paphiopedilum*.

**Figure 1.**
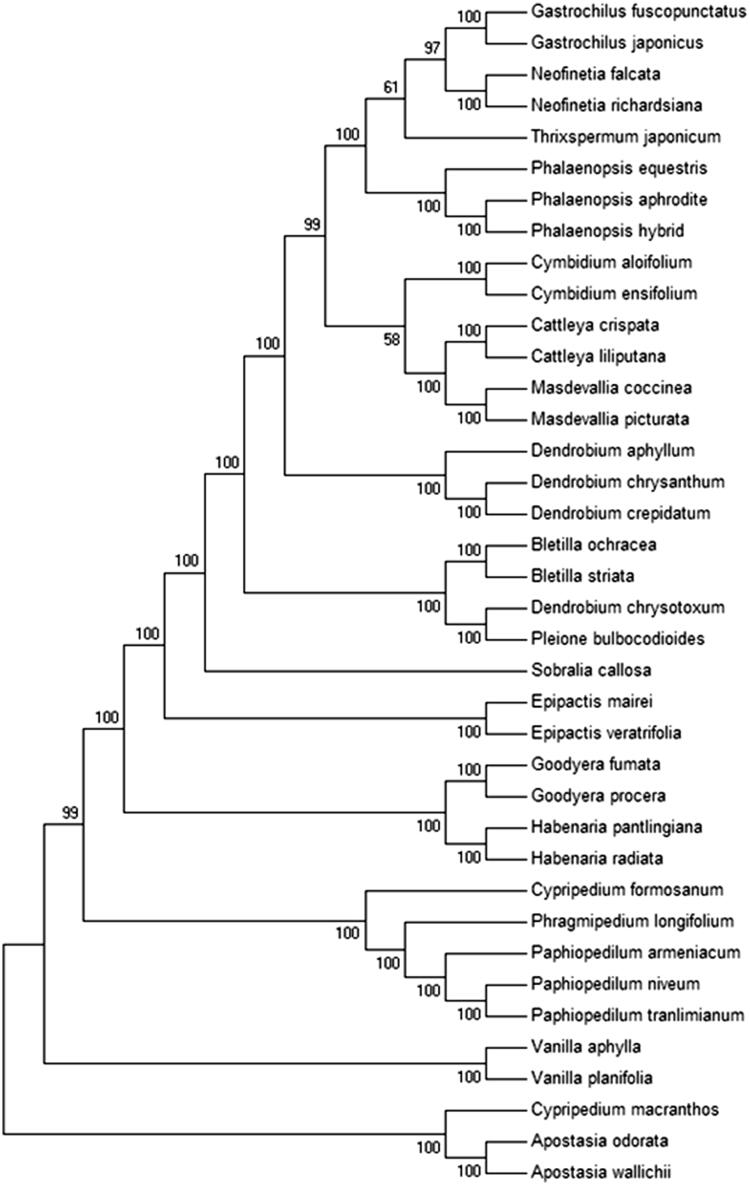
Phylogenetic of 38 species within the family Orchidaceae based on the Maximum-Likelihood analysis of the whole cp genome sequences using 500 bootstrap replicates. The analyzed species and corresponding Genbank accession numbers are as follows: Apostasia odorata(NC_030722.1), Apostasia wallichii(NC_036260.1), Bletilla ochracea(NC_029483.1), Bletilla striata(NC_028422.1), Cattleya crispata(NC_026568.1), Cattleya liliputana (NC_032083.1), Cymbidium aloifolium(NC_021429.1), Cymbidium ensifolium(NC_028525.1), Cypripedium formosanum(NC_026772.1), Cypripedium macranthos(NC_024421.1), Dendrobium aphyllum(NC_035322.1), Dendrobium chrysanthum(NC_035336.1), Dendrobium crepidatum(NC_035331.1), Epipactis mairei (NC_030705.1), Epipactis veratrifolia(NC_030708.1), Gastrochilus fuscopunctatus(NC_035830.1), Gastrochilus japonicus(NC_035833.1), Goodyera fumata(NC_026773.1), Goodyera procera(NC_029363.1), Habenaria pantlingiana(NC_026775.1), Habenaria radiata(NC_035834.1), Masdevallia coccinea(NC_026541.1), Masdevallia picturata(NC_026777.1), Neofinetia falcata(NC_036372.1), Neofinetia richardsiana(NC_036373.1), Paphiopedilum armeniacum(NC_026779.1), Paphiopedilum niveum(NC_026776.1), Phalaenopsis equestris(NC_017609.1), Phalaenopsis hybrid cultivar(NC_025593.1), Phragmipedium longifolium(NC_028149.1), Pleione bulbocodioides(NC_036342.1), Sobralia callosa(NC_028147.1), Thrixspermum japonicum(NC_035831.1), Vanilla aphylla(NC_035320.1), Vanilla planifolia(NC_026778.1).
